# A Self-Guided App-Based Mindfulness Intervention for Racially and Ethnically Minoritized Individuals Who Experience Discrimination-Related Mental Health Symptoms: Randomized Controlled Trial

**DOI:** 10.2196/84328

**Published:** 2026-05-14

**Authors:** Giovanni Ramos, Amanda K Montoya, Adrian Aguilera, Anna S Lau, Craig K Enders, Yinyin Wen, Denise A Chavira

**Affiliations:** 1 Department of Psychology, University of California, Berkeley, Berkeley, 2121 Berkeley Way, Berkeley, CA, 94704, United States, 1 5106425292; 2 Department of Psychology, University of California, Los Angeles, Los Angeles, CA, United States; 3 School of Social Welfare, University of California, Berkeley, Berkeley, CA, United States; 4 Department of Psychiatry and Behavioral Sciences, University of California, San Francisco, San Francisco, CA, United States

**Keywords:** digital mental health intervention, discrimination, mindfulness, racially and ethnically minoritized, stress, anxiety, depression, feasibility, equity, app

## Abstract

**Background:**

Racially and ethnically minoritized individuals (REMs) who experience discrimination are at risk of developing stress, anxiety, and depression, and digital mental health interventions (DMHIs) can make evidence-based treatments such as mindfulness available to these groups. However, REMs are significantly underrepresented in the overall DMHI and mindfulness-based DMHI literature, limiting our understanding of the effectiveness and feasibility of these digital tools to advance mental health equity for these populations.

**Objective:**

This randomized controlled trial evaluated the effectiveness of a 4-week self-guided app-based mindfulness DMHI versus treatment as usual (TAU) in reducing discrimination-related stress, anxiety, and depressive symptoms among REMs, while assessing feasibility metrics, including uptake, engagement, dropout, and program satisfaction.

**Methods:**

A total of 155 participants (mean age 27.28, SD 9.6 years) were randomized to either receive the DMHI (n=80) or TAU (n=75). Participants in the DMHI group underwent an onboarding procedure and were asked to complete 1 meditation daily for 4 weeks. Participants in the TAU group were encouraged to seek mental health services on their own. The racial and ethnic composition of the sample was 38.7% (60/155) Latinx, 37.4% (58/155) Asian, 11.6% (18/155) Black, 10.3% (16/155) Multiracial, and 2% (3/155) Native American. Mean differences between groups were examined using multilevel regression models, applying a false discovery rate (FDR) correction across all fixed effects. Treatment effect sizes and clinical significance were calculated using pseudo*-R^2^*s and the percentage of participants who achieved a minimal clinically significant difference (MCID), respectively. Feasibility outcomes were examined descriptively and included the percentage of participants who accessed the DMHI, days using it, meditations completed, total time meditated, the percentage of participants who dropped out of the study, and self-reported treatment satisfaction.

**Results:**

Compared with participants in the TAU group, participants in the DMHI group experienced greater reductions in stress (*β*=−4.52, 95% CI –6.54 to –2.51; FDR-adjusted *P*<.001; pseudo-*R*^2^=.41; MCID=61%, 49/80), anxiety (*β*=−3.31, 95% CI –4.89 to –1.74; FDR-adjusted *P*<.001; pseudo-*R*^2^=.45; MCID=48%, 38/80), and depression (*β*=−2.84, 95% CI –4.57 to –1.12; FDR-adjusted *P*=.004; pseudo-*R*^2^=.53; MCID=54%, 43/80) by the end of the program. All participants in the DMHI group downloaded the app (80/80, 100%) and used it, on average, 16.83 (SD 7.8) days, completed 24.85 (SD 23.3) mediations, and meditated 238.67 (SD 260.7) minutes. Of all participants, 12 (12/155, 8%) dropped out of this study. Most dropouts occurred in the intervention group (11/80, 14%). Participants perceived the DMHI program as satisfactory by the end of treatment (mean treatment satisfaction 23.14, SD 4.9).

**Conclusions:**

These findings position this self-guided app-based mindfulness DMHI as an effective and feasible strategy for mitigating the deleterious mental health consequences of exposure to discrimination among REMs.

## Introduction

In the United States, approximately 60% of racially and ethnically minoritized individuals (REMs) report experiencing daily instances of discrimination [1]. According to the Perceived Discrimination and Health Model [2], exposure to this chronic and unpredictable stressor triggers physiological (eg, cardiovascular reactivity), cognitive-affective (eg, rumination), and behavioral (eg, avoidance) responses that heighten the individual’s vulnerability to developing mental health problems. Exposure to discrimination is most strongly associated with increases in stress, anxiety, and depression symptoms [3]. This discrimination-related distress is likely to contribute to the high prevalence rates of these mental health problems in REMs [3,4]. While acknowledging that the most potent strategy to reduce this mental health burden is to dismantle the societal systems that perpetuate discrimination, there is still a need for individual-level interventions that could help REMs cope [5].

Among mental health treatments available to help REMs cope with discrimination-related mental health problems, mindfulness meditation may be particularly well-suited. In mindfulness meditation, practitioners cultivate present-moment awareness of their experience, including physical sensations, thoughts, and emotions, with an open and nonjudgmental attitude [6]. In the context of dealing with the mental health consequences of experiencing discrimination, mindfulness meditation reduces physiological reactivity, breaks repetitive negative thinking patterns, and promotes adaptive emotion regulation strategies, potentially disrupting some of the mechanisms by which discrimination “gets under the skin,” leading to stress, anxiety, and depression [5,7]. Numerous randomized controlled trials (RCTs) show that mindfulness interventions are effective in decreasing stress, anxiety, and depression [8]. However, mindfulness effectiveness research to date has been conducted with almost exclusively White samples [9], with only modest preliminary evidence in REMs [10].

Given the limited representation of REMs in mindfulness research, some researchers have theorized that, without careful cultural adaptation, these interventions may not be engaging or satisfying for these groups [11]. Concerns include incompatibility between REMs’ religious beliefs and the Buddhist origins of mindfulness, negative attitudes toward experiencing and expressing certain emotions, and perceived mismatches between collectivistic values and the individualistic nature of meditation. Trying to address these issues, mindfulness interventions have been culturally adapted for several REM groups [11-14]. However, whether those culturally adapted interventions are more effective than the original protocols remains an empirical question. In fact, one of the few meta-analyses examining the gains associated with culturally adapting mindfulness for REMs found that the number of cultural adaptations performed was not associated with treatment gains [14]. Nevertheless, these findings should be considered preliminary, given the small sample sizes in these studies, the heterogeneous changes implemented, and the lack of distinction between true cultural adaptations and implementation adaptations. Considering that culturally adapting interventions is a complex, time-consuming, and resource-intensive process, data on non–culturally adapted programs’ feasibility and effectiveness are needed to determine the need to, or lack thereof, make such adaptations [15].

Culturally adapted or not, REMs are less likely to access mindfulness interventions in the community [16]. These inequities in access seem to be driven mostly by provider shortages, transportation and time constraints, and treatment costs that disproportionally affect these groups [17]. Digital mental health interventions (DMHIs) could reduce this treatment gap by providing evidence-based treatments without the involvement of mental health professionals, making those interventions accessible anywhere at any time, and reducing costs [18]. Although in the United States REMs are still affected by the digital divide and are less likely than White individuals to have broadband internet at home [19], 85%‐96% of them still own a smartphone with internet access [20]. Furthermore, a growing body of evidence supports the effectiveness of smartphone-based DMHIs [21], including mindfulness apps [22]. However, like traditional mindfulness interventions, there is limited representation of REMs in DMHI research [23], including mindfulness-based DMHIs [22].

Including REMs in DMHI research is necessary to determine whether these approaches are truly able to reduce inequities in mental health. For instance, in mindfulness-based DMHI RCTs, up to 55% of participants never access the app, and those who do so only use it a couple of times before approximately 25% drop out of treatment [24]. These rates are even higher in real-world app usage, with up to 79% of users never opening the mental health app even after downloading it [25] and only 4% of those who downloaded it continue using it after 2 weeks [26]. Considering that REMs face numerous barriers to engaging with traditional mental health services [17], it may be possible that they also face significant difficulties engaging in DMHIs. Indeed, not having enough time, forgetting to use the digital tool, a lack of motivation, skepticism about the need for treatment and treatment effectiveness, and cost were the most commonly reported barriers to using DMHIs among REMs [27,28]. Therefore, examining the uptake of, engagement with, dropout in, and program satisfaction with DMHIs among REMs is a pressing need in the field.

Considering the significant gaps in both traditional and DMHI mindfulness research with REM groups, this RCT examines the effectiveness and feasibility of a self-guided app-based mindfulness DMHI for REMs who experience elevated levels of discrimination.

Aim 1: Test the effectiveness of a mindfulness-based DMHI in reducing stress, anxiety, and depression among REMs who experience elevated levels of discrimination. Hypothesis 1: Based on results from traditional mindfulness interventions [8] and mindfulness-based DMHIs [22], we hypothesized that participants in the DMHI group would experience greater reductions in stress, anxiety, and depression compared with their peers in the treatment-as-usual (TAU) group.

Aim 2: Describe the feasibility of this DMHI program, including its uptake, engagement, dropout, and self-reported program satisfaction. Hypothesis 2: Given the lack of consensus on the most important engagement metrics in DMHIs [29] and benchmarks for what constitutes “optimal” engagement with these tools [30], we did not have specific hypotheses.

## Methods

### Study Context

This parallel-group RCT took place at the intersection of 3 major political and social events in the United States from 2020 to 2022 that made discrimination against REMs especially salient. First, a hostile political discourse against REMs, which was associated with increases in discrimination against these groups [31]. Second, mental health problems caused by exposure to discrimination among REMs who were blamed for the COVID-19 pandemic [32]. Third, multiple instances of police violence against REMs, which were associated with poor mental health outcomes at the population level [33]. Considering this context, there was a significant need for mental health services that could help REMs cope with symptoms of stress, anxiety, and depression associated with experiencing discrimination.

### Ethical Considerations

The University of California, Los Angeles (UCLA) Institutional Review Board approved all procedures in this study (IRB 21‐001426). The study hypotheses and data analytic plan were preregistered (NCT05027113), and the study protocol was peer-reviewed and published as a registered report (DERR1-10.2196/35196) [31]. We report any deviations from the registered report in each pertinent section. While this study focuses on the main clinical and feasibility outcomes of this RCT, additional measures of mechanisms of action were collected and will be reported in future publications. There are no previous publications coming from this dataset. A member of the research team contacted individuals who met eligibility criteria over the phone to discuss the study, answer questions, and obtain their informed consent to participate. Electronic raw data were stored in the Health Insurance Portability and Accountability Act (HIPAA)-compliant UCLA’s Qualtrics server. Data were deidentified by assigning each participant a unique ID number and keeping consent forms and other digital documents containing identifiable information separate from study data. All participants received a free 2-month subscription to the DMHI as an incentive to participate. Additionally, participants were compensated with US $10, US $15, and US $25 for completing questionnaires and sharing app usage data at baseline, midtreatment, and posttreatment, respectively. Reporting in this trial adhered to the Consolidated Standards of Reporting Trials of Electronic and Mobile Health Applications and Online Telehealth (CONSORT-EHEALTH) [34].

### Procedure

Nationwide recruitment took place from October 2021 to April 2022. Recruitment materials depicted REMs and used language that emphasized the stressful nature of experiencing discrimination. Materials were posted on social media, emailed to relevant social and professional groups, and physically distributed in communities with a high representation of REMs in Los Angeles, California. Individuals interested in participating in the study completed an online screening questionnaire. For study inclusion, participants were required to (1) identify as a REM, (2) consider themselves fluent in English, (3) not receive psychological services currently, (4) not have practiced mindfulness meditation for more than 2 hours in the month prior to study commencement, (5) own a smartphone with access to the internet, (6) be willing to install the mindfulness app and receive daily notifications and text reminders, (7) be at least 18 years old, and (8) report experiencing elevated levels of discrimination (ie, Multicultural Discrimination Module [MDM] score ≥12 [35]). Originally, participants who scored 55 or greater on the Index of Race-Related Stress-Brief (IRRS-B) [36] were also eligible. However, both measures seemed redundant as evidenced by their high correlation (*r*=0.72) and virtually equivalent correlations with the clinical outcomes at baseline—stress (*r*=0.24 and *r*=0.26), anxiety (*r*=0.60 and *r*=0.60), and depression (*r*=0.73 and *r*=0.73). Considering also that the MDM was developed for multiple REM groups while the IRRS-B was originally intended for one specific REM group (ie, Black), and the better psychometric properties of the MDM (ω=.93) compared with the IRRS-B (ω=.71) seen in this sample, we opted to only use the MDM inclusion criterion in this study. Nevertheless, this decision did not affect the final number of participants deemed eligible, enrolled, or included in the analyses.

Participants were randomly assigned to either the DMHI (n=80) or TAU (n=75) condition using computer-generated block randomization with a fixed block size of 5. The randomization sequence was generated automatically, and allocation was concealed until assignment. Due to the nature of the intervention, participants were not blinded to condition assignment. However, masking occurred at the levels of care provider (ie, the intervention was delivered via a fully automated DMHI), investigators (ie, group assignment was determined by a computer program without investigators’ involvement), and outcome assessment (ie, all outcomes were collected via self-administered online assessments). Participants randomized to the DMHI group underwent a short, standardized onboarding procedure over the phone (approximately 30 min) in which they were assisted with installing the app, learning to use it, and setting reminders and notifications. Participants in the TAU condition were reminded of the scheduled assessments, encouraged to seek mental health services or use a DMHI on their own if interested, and given access to the study’s DMHI 4 weeks after randomization. Data were collected entirely through self-assessments online at baseline (ie, randomization or wk 0), midtreatment (ie, wk 2), and posttreatment (ie, wk 4).

### Group Conditions

#### DMHI Group

The Mindfulness for Us, or Mind-Us for short, program consisted of using the commercially available Ten Percent Happier app (now Happier Meditation) to complete at least 1 meditation daily, with the possibility of exceeding this goal by choosing additional meditations, for 4 weeks. More specifically, participants were asked to complete “The Basics,” “The Basics II,” and “Essential Advice” courses to learn the basics of mindfulness meditation. Each session begins with a short video providing psychoeducation on mindfulness principles (eg, awareness of the present moment) followed by teaching a specific mindfulness technique (eg, noting thoughts and emotions). These introductory courses also dispel common myths about mindfulness meditation (eg, meditators empty their minds) and teach users how to deal with common meditation obstacles (eg, strong negative emotions). The courses comprise 29 sessions, in which the meditation length gradually increases from 5 to 18 minutes. For additional practice, participants were allowed to select any meditation from the app. We chose this mindfulness app, given its high user ratings (ie, 4.8 stars out of 5 in app stores) and previous evidence of effectiveness with mostly White samples [37,38].

We chose to use a commercially available app instead of a culturally adapted one, given evidence showing that DMHIs based on evidence-based principles are effective for REMs, regardless of whether they are culturally adapted or not [15,18]. This approach has the potential to advance mental health equity for REM by making existing DMHIs immediately available to these groups, rather than waiting for culturally adapted versions to be developed [15]. Furthermore, using commercially available DMHIs with REMs can allow researchers to document potential areas of improvement for these tools, effectively gathering the data necessary to inform data-driven cultural adaptations of DMHIs that are more likely to improve treatment outcomes than when assuming a priori that these adaptations are needed [15,18,39].

In an effort to minimize engagement issues commonly observed in self-guided app-based DMHIs [26,40], the Mind-Us program used a combination of engagement-enhancement strategies. First, participants were encouraged to substitute longer meditations with shorter ones as needed to make meditation fit their routine and facilitate daily practice. The Ten Percent Happier app also allowed users to set a daily notification that can be personalized. Similarly, participants received 3 daily text messages in the morning, noon, and evening with psychoeducational content, prompts encouraging them to reflect on their progress, and reminders to meditate and complete study assessments. During the onboarding call, study staff also helped participants generate their own strategies to deal with potential barriers to treatment engagement (eg, integrating the use of the app into daily routines such as before going to bed).

Following recommendations on how to improve the cultural fit of mindfulness-based interventions [5,11-14] and DMHIs for REMs [18], during onboarding participants also discussed any reservations about using an app not explicitly designed for REM groups and received psychoeducation about how mindfulness meditation could help them cope with the mental health consequences of experiencing discrimination. In this discussion, study staff emphasized that mindfulness meditation would not teach participants to “accept” or “reappraise” the discriminatory behavior of others but rather to gain awareness of the distress associated with the experience, validate their emotional responses, and gain distance between negative messages about REM groups and their own sense of self [5]. All participants in the DMHI group (80/80, 100%) completed this onboarding procedure.

#### TAU Group

Originally, we had proposed to use a waitlist group as the control condition. Instead, participants randomized to the control group were encouraged to seek professional mental health services or use commercially available DMHIs on their own, verified via self-report at midtreatment and posttreatment, effectively making this control condition TAU. Given our overarching goal of making mental health services available to REMs exposed to discrimination, participants in the TAU group were also given access to the DMHI after completing the postintervention assessment. Then, they received an email with the same instructions for downloading and using the app that participants in the intervention group received during the onboarding call. We chose this control condition following the Pragmatic Model for Comparator Selection in Health-Related Behavioral Trials framework [41], which indicated that, given the limited effectiveness evidence for both face-to-face [8,10] and DMHI-based mindfulness programs for REM groups [22], a TAU control was adequate.

### Measures

#### Screening

##### Discrimination

The 8-item MDM [35] is a measure of discrimination for different racial, ethnic, and cultural groups. Participants rate items on a scale ranging from 1 (never) to 4 (often), with higher scores indicating higher levels of discrimination over the last year. Items include “How often have you been treated with less respect than other people” and “How often have you been threatened or harassed.” The MDM was developed using a nationally representative sample of REMs in the United States and has shown validity and good internal consistency (α=0.81-0.88) [35]. For study inclusion, participants needed to obtain at least a score of 12 at screening, which falls within the 75th percentile of the population distribution scores. In this study, the MDM reliability was excellent (ω=.93).

### Effectiveness Outcomes

#### Stress

The 10-item Perceived Stress Scale (PSS) [42] is a measure of perceived stress. Participants rate items on a scale ranging from 0 (never) to 4 (very often), with higher scores indicating more severe stress symptoms over the last 2 weeks. The measure includes items, such as “How often have you felt that you were unable to control the important things in your life?” and “How often have you felt confident about your ability to handle your personal problems?” The PSS has shown good reliability (α=0.84-0.86) and validity with REM samples [43]. The PSS was administered at baseline, midtreatment, and posttreatment. In this study, the PSS reliability was excellent (ω=.87-.91).

#### Anxiety

The 7-item General Anxiety Disorder-7 (GAD-7) [44] is a measure of anxiety symptoms. Participants rate items on a scale ranging from 0 (not at all) to 3 (nearly every day), with higher scores indicating more severe anxiety symptoms over the last 2 weeks. The scale includes items, such as “Feeling nervous, anxious or on edge,” and “Not being able to stop or control worrying.” The GAD-7 has shown good internal consistency (α=0.89-0.90) and validity with REM samples [45]. The GAD-7 was administered at baseline, midtreatment, and posttreatment. In this study, the GAD-7 reliability was excellent (ω=.88-.93).

#### Depression

The 8-item Patient Health Questionnaire-8 (PHQ-8) is a measure of depressive symptoms derived from the original 9-item Patient Health Questionnaire-9 (PHQ-9) [46], which omits the suicidal ideation item while still being virtually equivalent [47]. Participants rate items on a scale ranging from 0 (not at all) to 3 (nearly every day), with higher scores indicating more severe depressive symptoms over the last 2 weeks. This measure includes items, such as “Little interest or pleasure in doing things” and “Feeling down, depressed, or hopeless.” The PHQ-8 has shown good reliability (α=0.86-0.89) and validity across different racial and ethnic groups [48]. The PHQ-8 was administered at baseline, midtreatment, and posttreatment. In this study, the PHQ-8 reliability was excellent (ω=.87-.90).

### Feasibility Outcomes

#### Uptake

This feasibility outcome was operationalized as the percentage of participants who downloaded the app and created a user profile during the 4-week intervention.

#### Engagement

The number of days using the app, the number of meditations completed, and the total time meditated in minutes were used as behavioral measures of DMHI engagement. These implementation outcomes were tracked by the DMHI. Participants were asked to share their app usage data via screenshots when completing midtreatment and posttreatment assessments. Previous studies have shown that this method provides accurate behavioral indicators of treatment engagement [37,49].

#### Dropout

This feasibility outcome was operationalized as the percentage of participants who left the program and did not complete the posttreatment assessment, regardless of their level of engagement with the DMHI.

#### Program Satisfaction

The 6-item Satisfaction with Therapy subscale of the Satisfaction with Therapy and Therapist Scale-Revised (STTS-R) [50] was used to assess program satisfaction with the DMHI program. Participants rate items on a scale ranging from 1 (strongly disagree) to 5 (strongly agree), with higher scores indicating more program satisfaction in the last 2 weeks. Scores above 18 on this subscale are considered satisfactory. Examples of items include “I am satisfied with the quality of the program I received” and “I would recommend this program to a friend.” The Satisfaction with Therapy subscale has shown excellent reliability (α=0.92) and validity in DMHIs with REM groups [51]. The Satisfaction with Therapy subscale was administered at midtreatment and posttreatment. In this study, the Satisfaction with Therapy subscale reliability was excellent (ω=.91-.93).

### Data Analytic Plan

The original preregistered power analysis was updated based on more recent estimates of the treatment effect sizes of app-based mindfulness DMHIs [22] and published test-retest estimates for repeated measures of stress [43], anxiety [52], and depression [53]. A power analysis using G*Power (version 3.1.9.6; Faul, Erdfelder, Lang, and Buchner) [54] indicated that, based on 2 groups, 3 repeated measurements, a 2-week correlation of *r*=.60, and an average treatment effect size of *d*=0.30 (range 0.24‐0.42), a sample of n=94 would provide 80% power to detect potential group-by-time interactions. Expecting an approximate 25% dropout rate similar to previous app-based mindfulness DMHI trials [24], we originally planned to recruit 120 participants. However, given the high demand for this program (ie, more than 50% of the original sample was recruited within the first month of study commencement), we expanded recruitment until resources to provide financial compensation to participants were exhausted.

All statistical analyses were conducted using R (version 4.3.2; R Core Team) [55]. Model assumptions were evaluated, including the distribution of outcomes, linearity of relationships, residual normality and homoscedasticity, and multicollinearity among predictors. Inspection of diagnostic plots and indices indicated no apparent violations, supporting the adequacy of the specified models. To test mean differences between groups, we conducted multilevel regression models with group condition as the predictor, time as a within-group factor, and a group-by-time interaction. We examined potential group differences from baseline to posttreatment (ie, wk 0 to wk 4), baseline to midtreatment (ie, wk 0 to wk 2), and midtreatment to posttreatment (ie, wk 2 to wk 4). We included participant age, gender, and perceived financial stress as covariates in all analyses, considering previous studies with REM populations showing differences in the frequency and impact of discrimination on mental health outcomes [1,2], as well as differences in the prevalence of mental health problems based on these demographics [4]. We fitted all models using the *nlme* package in R (version 3.1-158; R Core Team) [56], including random intercepts, an autoregressive error structure, and restricted maximum likelihood as the estimation method. To control for multiple testing in each model, we applied a Benjamini-Hochberg false discovery rate (FDR) correction across all fixed effects using the *p.adjust* function in R (version 4.3.2; R Core Team). This type I correction included all predictors and covariates, which provided a more conservative control of false discoveries by accounting for all inferential tests that could influence model interpretation. To determine the clinical significance of treatment effects, we compared reductions in the PSS, GAD-7, and PHQ-8 scores observed in this study with the minimum clinically important difference (MCID) established for these measures. The MCID represents the smallest clinically relevant reduction in symptoms, regardless of whether the difference is statistically significant [57]. Previous studies have established MCIDs of 4 points for the PSS [58] and 3 points for both the GAD-7 and PHQ-9 [59]. Although the PHQ-8 and PHQ-9 are virtually equivalent [47], we prorated the PHQ-8 scores by multiplying them by 1.125 to align them with the PHQ-9 score range and facilitate comparisons with previous studies and future meta-analytic work. This is a common practice in DMHI studies using the PHQ-8 [60]. Nonprorated results were virtually equivalent and are publicly available elsewhere [61]. To determine treatment effect sizes, we calculated pseudo-*R^2^* statistics for all models using the *r2mlm* package in R (version 0.3.2; R Core Team) [62], which provides estimates of the total variance explained by the model. We also examined the feasibility outcomes of this DMHI (ie, uptake, engagement, dropout, and program satisfaction) using descriptive statistics. Originally, we had proposed to use the last-observation-carried-forward method to handle missing data. Instead, we tested the robustness of these results under a variety of missing data models and assumptions about the missing data mechanisms, using a Bayesian latent variable estimation modeling and imputation approach using Blimp (version 4.0; Keller and Enders) [63]. Only 8% (13/155) of participants had missing outcome data in this trial. Similarly, only 12% (9/80) of participants had missing engagement data in the DMHI group. None of the results changed based on the missing data model used. Therefore, we present results using an imputed dataset following the model with the best performance (Multimedia Appendix 1). Finally, we opted not to include per-protocol analyses as originally planned. Instead, we only conducted and reported intention-to-treat analyses to provide a more accurate estimate of DMHI effectiveness.

## Results

**Figure 1.**
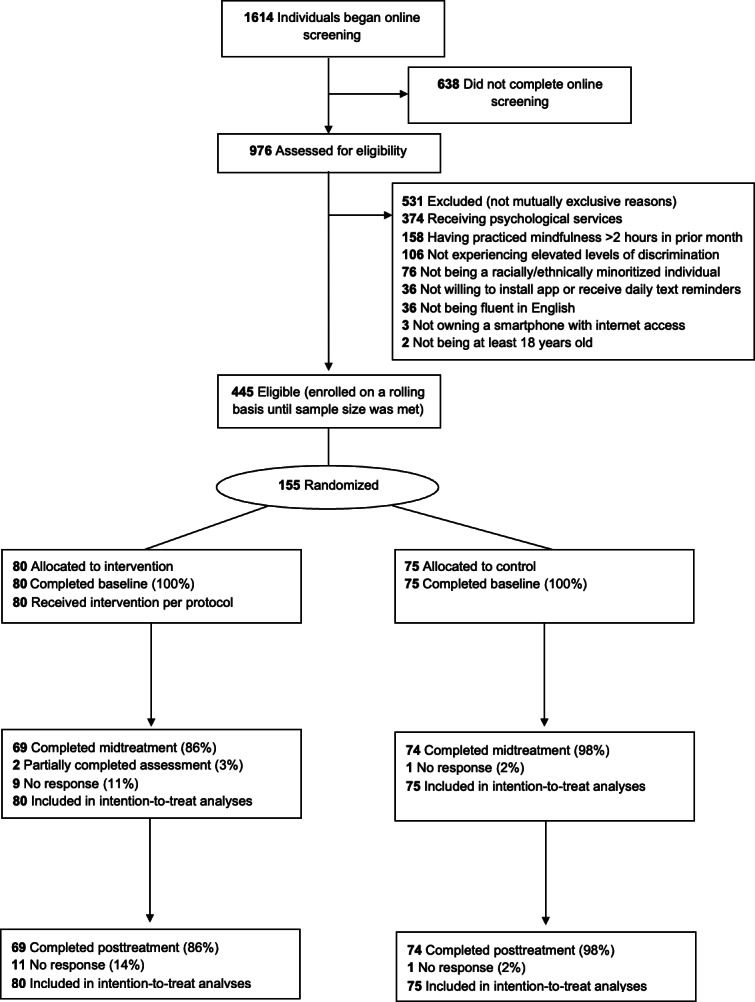
Study design flowchart.

The flowchart of participants’ recruitment, allocation, and analysis is presented in Figure 1.

Table 1 shows the demographics of the final sample (N=155).

**Table 1.** Baseline characteristics of participants in the TAU^a^ group, DMHI^b^ group, and total sample.

Variable	TAU (n=75)	DMHI (n=80)	Total (N=155)
Age (years), mean (SD)	27.49 (10.1)	27.09 (9.2)	27.28 (9.6)
Race and ethnicity, n (%)			
Latinx	29 (38.7)	31 (38.8)	60 (38.7)
Asian	28 (37.3)	30 (37.5)	58 (37.4)
Black	9 (12)	9 (11.2)	18 (11.6)
Multiracial	7 (9.3)	9 (11.2)	16 (10.3)
Native American	2 (2.7)	1 (1.3)	3 (2)
Gender, n (%)			
Female	59 (78.6)	69 (86.2)	128 (82.6)
Male	11 (14.7)	11 (13.8)	22 (14.2)
Nonbinary	5 (6.7)	0 (0)	5 (3.2)
Educational level, n (%)			
Less than high school	0 (0)	1 (1.3)	1 (0.6)
High school or General Educational Development	10 (13.3)	4 (5)	14 (9)
Trade school	0 (0)	1 (1.3)	1 (0.6)
Some college, no degree	18 (24)	27 (33.8)	45 (29)
College	21 (28)	25 (31.2)	46 (29.8)
Graduate degree	26 (34.7)	22 (27.4)	48 (31)
Occupation, n (%)			
Unemployed	1 (1.3)	2 (2.5)	3 (2)
Employed	27 (36)	38 (47.5)	65 (41.9)
Student	46 (61.4)	40 (50)	86 (55.5)
Retired	1 (1.3)	0 (0)	1 (0.6)
Relationship status, n (%)			
Single	45 (60)	43 (53.7)	88 (56.8)
In a long-term relationship	27 (36)	36 (45)	63 (40.6)
Separated or divorced	3 (4)	1 (1.3)	4 (2.6)
Financial stress, n (%)			
Yes	14 (18.7)	10 (12.5)	24 (15.5)
No	61 (81.3)	70 (87.5)	131 (84.5)
Previous mindfulness experience, n (%)			
Yes	33 (44)	41 (51.2)	74 (47.7)
No	42 (56)	39 (48.8)	81 (52.3)
MDM^c^, mean (SD)	19.29 (4.7)	19.25 (4.6)	19.27 (4.6)
PSS^d^, mean (SD)	20.15 (6)	21.55 (5)	20.87 (5.5)
GAD-7^e^, mean (SD)	7.91 (4.5)	9.12 (4.6)	8.53 (4.6)
PHQ-9^f^^,^^g^, mean (SD)	8.96 (5.5)	9.95 (5.5)	9.4 (5.5)

a TAU: treatment as usual.

b DMHI: digital mental health intervention.

c MDM: Multicultural Discrimination Module.

d PSS: Perceived Stress Scale.

e GAD-7: General Anxiety Disorder-7.

f PHQ-9: Patient Health Questionnaire-9.

g PHQ-8 scores were prorated by multiplying them by 1.125 such that they were in the same score range as those in the PHQ-9.

**Figure 2.**
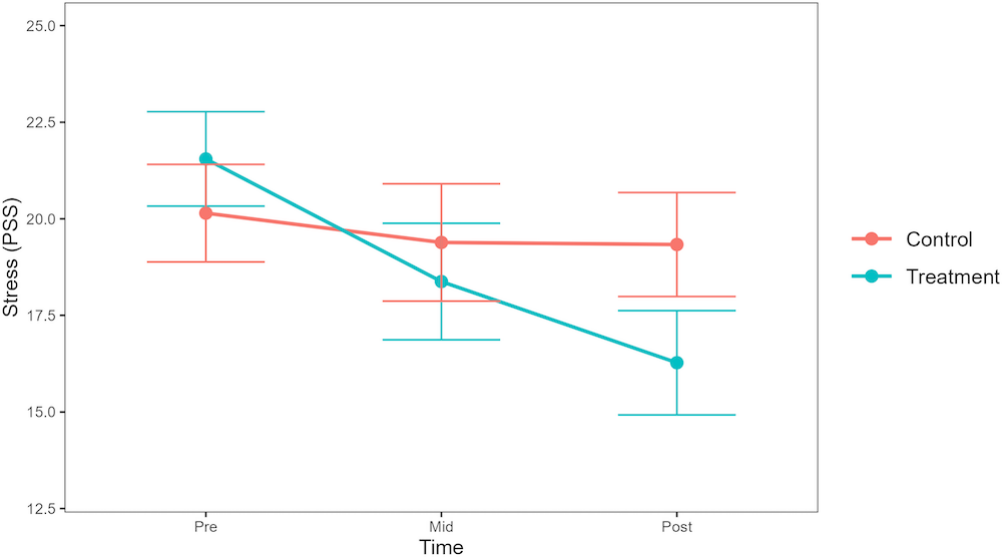
Trajectories of change in stress symptoms across groups. PSS: Perceived Stress Scale.

In the model for stress, there was a significant group-by-time interaction showing a greater decrease in stress among participants in the DMHI group compared with participants in the TAU group at posttreatment (*β*=−4.52, 95% CI −6.54 to−2.51, SE 1.03, *t*_7511_=−4.40; FDR-adjusted *P*<.001). By posttreatment, 61% (49/80) of participants in the DMHI group achieved the 4-point MCID reduction, compared with 23% (17/75) in the TAU group. As seen in Figure 2, these differences in change over time emerged from baseline to midtreatment (*β*=−2.41, 95% CI −4.14 to −0.67, SE 0.89, *t*_10441_=−2.72; FDR-adjusted *P*=.01; MCID in DMHI group=50%, 40/80, MCID in TAU group=29%, 22/75) and continued from midtreatment to posttreatment (*β*=−2.11, 95% CI −3.90 to –0.32, SE 0.91, *t*_1977_=−2.32; FDR-adjusted *P*=.03; MCID in DMHI group=37%, 30/80, MCID in TAU group=21%, 16/75). The total variance (pseudo-*R*^2^) explained by the model was 0.41.

**Figure 3.**
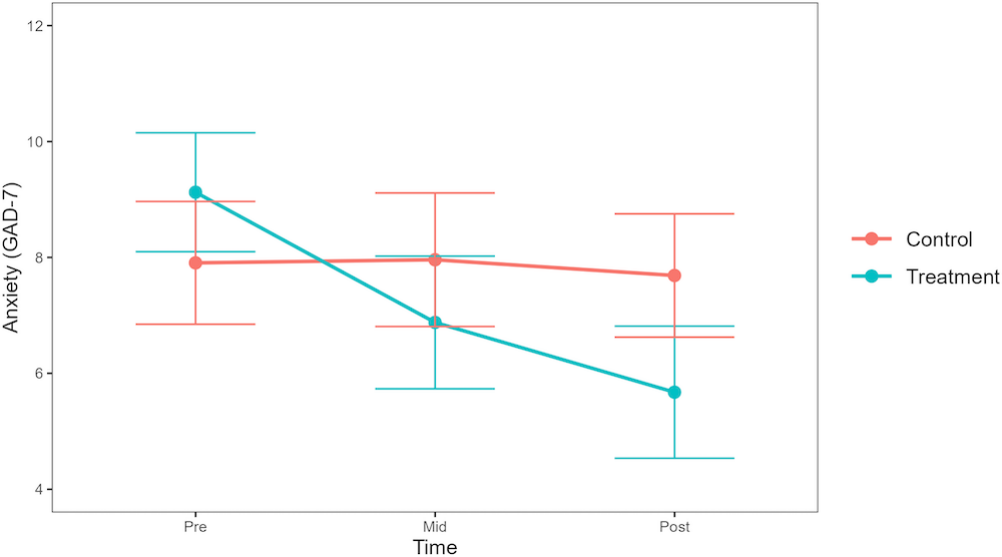
Trajectories of change in anxiety symptoms across groups. GAD-7: General Anxiety Disorder-7.

In the model for anxiety, there was a significant group-by-time interaction showing a greater decrease in anxiety among participants in the DMHI group compared with participants in the TAU group at posttreatment (*β*=−3.31, 95% CI −4.89 to −1.74, SE 0.80, *t*_22128_=−4.13; FDR-adjusted *P*<.001). By posttreatment, 48% (38/80) of participants in the DMHI group achieved the 3-point MCID reduction, compared with 27% (20/75) in the TAU group. As seen in Figure 3, these differences in change over time emerged from baseline to midtreatment (*β*=−2.31, 95% CI −3.77 to –0.85, SE 0.75, *t*_6191_=−3.10; FDR-adjusted *P*=.005; MCID in DMHI group=50%, 40/80, MCID in TAU group=22%, 17/75), and the reduction in symptoms continued the same trajectory from midtreatment to posttreatment but without reaching statistical significance (*β*=−1.00, 95% CI −2.47 to 0.47, SE 0.75, *t*_4626_=−1.33; FDR-adjusted *P*=.25; MCID in DMHI group=30%, 24/80, MCID in TAU group=22%, 17/75). The total variance (pseudo-*R*^2^) explained by the model was 0.45.

**Figure 4.**
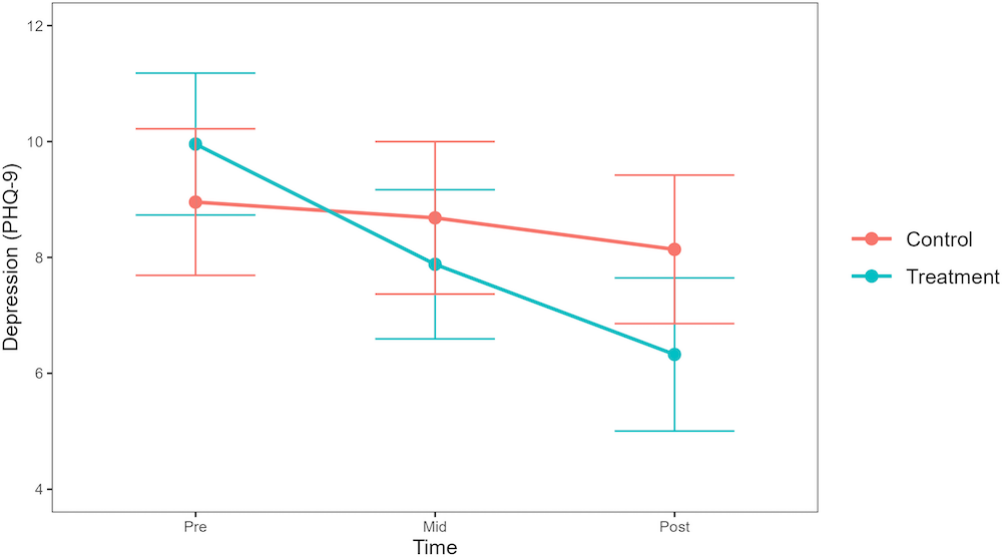
Trajectories of change in depressive symptoms across groups. PHQ-9: Patient Health Questionnaire-9.

In the model for depression, there was a significant group-by-time interaction showing a greater decrease in depression among participants in the DMHI group compared with participants in the TAU group at posttreatment (*β*=−2.84, 95% CI −4.57 to −1.12, SE 0.88, *t*_11660_=−3.23; FDR-adjusted *P*=.004). By posttreatment, 54% (43/80) of participants in the DMHI group achieved the 3-point MCID reduction, compared with 31% (23/75) in the TAU group. As seen in Figure 4, these differences in change over time emerged from baseline to midtreatment (*β*=−1.86, 95% CI −3.38 to –0.33, SE 0.78, *t*_8235_=−2.38; FDR-adjusted *P*=.04; MCID in DMHI group=46%, 37/80, MCID in TAU group=24%, 18/75), and the reduction in symptoms continued the same trajectory from midtreatment to posttreatment but without reaching statistical significance (*β*=−0.99, 95% CI −2.52 to 0.54, SE 0.78, *t*_7566_=−1.26; FDR-adjusted *P*=.24; MCID in DMHI group=39%, 31/80, MCID in TAU group=30%, 23/75). The total variance (pseudo-*R*^2^) explained by the model was 0.53. Table 2 shows descriptive statistics of clinical outcomes at each assessment point.

**Table 2.** Descriptive statistics of clinic outcomes with missing data imputed.

Outcome and group	Baseline, mean (SD)	Midtreatment, mean (SD)	Posttreatment, mean (SD)
Stress (PSS)^a^ (range 0‐40)			
Control	20.15 (6)	19.39 (6.3)	19.33 (6.1)
Intervention	21.55 (5)	18.38 (6.8)	16.28 (5.6)
Whole sample	20.87 (5.5)	18.87 (6.6)	17.76 (6)
Anxiety (GAD-7)^b^ (range 0‐21)			
Control	7.91 (4.5)	7.96 (5.3)	7.69 (5)
Intervention	9.12 (4.6)	6.88 (4.7)	5.67 (4.2)
Whole sample	8.54 (4.6)	7.40 (5)	6.65 (4.7)
Depression (PHQ-9)^c^^,d^ (range 0‐27)			
Control	8.96 (5.5)	8.68 (5.8)	8.14 (5.9)
Intervention	9.96 (5.5)	7.88 (5.5)	6.33 (5.2)
Whole sample	9.47 (5.5)	8.27 (5.7)	7.20 (5.6)

a PSS: Perceived Stress Scale.

b GAD-7: Generalized Anxiety Disorder-7.

c PHQ-9: Patient Health Questionnaire-9.

d PHQ-8 scores were prorated by multiplying them by 1.125 such that they were in the same score range as those in the PHQ-9.

In terms of feasibility, all participants in the DMHI group (80/80, 100%) downloaded the app and created a user profile. By the end of the 4-week program, participants used the app for a mean of 16.83 (SD 7.8; range 0‐28) days, completed a mean of 24.85 (SD 23.3; range 0‐28) meditations, and meditated for a mean of 238.67 (SD 260.7; range 0‐2195) minutes. Only 2% of participants (2/80) did not use the DMHI at all. Participants used the app for a mean of 9.01 (SD 3.6; range 0‐14) days, completed a mean of 13.27 (SD 7.7; range 0‐52) meditations, and meditated for a mean of 113.76 (SD 90.5; range 0‐652) minutes from baseline to midtreatment. During these first 2 weeks of the program, 2% of participants (2/80) did not use the DMHI at all. Participants also used the app for a mean of 7.85 (SD 4.7; range 0‐14) days, completed a mean of 11.58 (SD 17.2; range 0‐156) meditations, and meditated a mean of 124.90 (SD 179.7; range 0‐1543) minutes from midtreatment to posttreatment. During these last 2 weeks of the program, 7% of participants (6/80) did not use the DMHI at all. Only 8% of participants (12/80) dropped out of this study. Of note, 11 of them (11/80, 14%) came from the DMHI group. In terms of program satisfaction, participants reported being satisfied with the DMHI program at both posttreatment (program satisfaction mean 23.14, SD 4.9) and midtreatment (program satisfaction mean 21.97, SD 5.1). No participant in the TAU group (0/80, 0%) reported accessing professional mental health services or a commercially available DMHI during the 4-week program.

## Discussion

### Principal Findings

The goal of this study was to examine the effectiveness and feasibility of a self-guided app-based DMHI for REMs who experience significant levels of discrimination. Results showed that the Mind-Us program was effective in reducing symptoms of stress, anxiety, and depression in as little as 2 weeks. Crucially, all participants assigned to the intervention group accessed the DMHI, engagement was consistent throughout 4 weeks, the dropout rate was low, and participants perceived the program as satisfactory. Taken together, these findings suggest that DMHIs can target the unique mental health needs of REMs. As such, low-intensity DMHI approaches hold significant promise to provide REMs with evidence-based treatments to cope with the deleterious mental health consequences of experiencing discrimination.

Based on results from 158 studies of traditional mindfulness interventions [8,10] and 43 studies of mindfulness-based DMHIs [22], this RCT is one of the first studies to support the effectiveness of a low-intensity mindfulness-based DMHI in reducing discrimination-related mental health symptoms across multiple REM groups. Consistent with our hypothesis, REMs who received the DMHI experienced significant short-term reductions in stress, anxiety, and depression. Although not in the context of experiencing discrimination and mostly with White samples, these results align with previous mindfulness-based DMHIs, showing these interventions are effective in targeting these mental health problems [22]. Notably, these reductions in stress, anxiety, and depression are not only statistically significant but also clinically meaningful. By the end of treatment, 61%, 48%, and 54% of participants experienced a decrease in stress, anxiety, and depression, respectively, that can be considered clinically significant according to empirically established cutoffs [58,59]. Considering that most mindfulness-based DMHI trials to date have failed to examine whether treatment effects translate into meaningful clinical changes [22], this study contributes to the mindfulness and DMHI literatures by demonstrating that this approach is effective for REMs exposed to discrimination. Taken together, these results help establish self-guided mindfulness-based DMHIs as a potentially effective approach to help REM groups cope with mental health problems associated with being exposed to discrimination.

Another important finding was that the Mind-Us program led to significant symptom reductions even after only 2 weeks of treatment. Most mindfulness-based DMHIs to date have examined high-intensity programs lasting 8 weeks or longer, requiring intensive meditation practice, or including some contact with a mindfulness facilitator [22]. In contrast, this DMHI yielded significant improvement in symptoms after 2 weeks of using the mindfulness app for less than 10 minutes a day and without the support of a mindfulness facilitator. Further, these reductions in stress, anxiety, and depressive symptoms were clinically meaningful for approximately 50% of participants who received the DMHI by MCID benchmarks [58,59]. This treatment response pattern is consistent with other mindfulness-based DMHIs, showing that most of the clinical gains seem to be made in the first 10 days of treatment [64]. This treatment response is especially encouraging as DMHIs that involve a low time commitment may be able to engage vulnerable populations who are unable to participate in more intensive mental health services due to competing demands and logistical barriers.

Even if participants experienced significant treatment gains in the first 2 weeks, the Mind-Us program led to even greater reductions in symptoms after 4 weeks of treatment, which suggests that additional mindfulness practice is important to promote further improvement. An important caveat is that, except for stress symptoms that continued to decrease, participants did not experience significant reductions in anxiety or depressive symptoms from midtreatment to posttreatment (ie, from wk 2 to wk 4). As previously discussed, other mindfulness-based DMHIs have found that improvements in symptoms occur at a slower rate after 10 days of treatment [64]. The difference in treatment response for stress symptoms versus anxiety and depressive symptoms could be due to the nature of these mental health problems. Stress symptoms seem to be more short-lived and situational compared with anxiety and depressive symptoms that are more pervasive and persistent [65]. Therefore, stress may be a more modifiable clinical target in DMHIs. In fact, mindfulness-based DMHIs often lead to larger treatment effect sizes when targeting stress compared with anxiety or depression [8,66]. Together, our findings suggest that there may be varying durations of mindfulness-based DMHIs that could be beneficial for REMs, especially based on the clinical target (ie, stress vs anxiety or depression). Future work still needs to determine the optimal mindfulness dosage in DMHIs that maximizes symptom reduction while maintaining the tolerability of the program, whether dosage optimization differs across subgroups (eg, different REM groups and individuals with different symptomatology profiles), and potential dose-response interactions.

Notably, the Mind-Us program used a commercially available mindfulness app not designed specifically for REMs, suggesting that non–culturally adapted DMHIs can be effective and well-accepted among these groups. Showing proof of DMHI effectiveness and program satisfaction among REM groups is essential, considering ongoing concerns about potential mismatches between mindfulness interventions and the cultural background of REMs [11], the lack of DMHI content that responds to the unique needs of REMs [67], and the need (or not) to culturally adapt DMHIs [15,18]. The results of this trial support using DMHIs with a strong evidence base and high treatment satisfaction profiles, even if not previously established for specific REM populations. This is an approach that can advance mental health equity more immediately than waiting for REM-specific DMHIs to be developed [15,39]. While DMHIs often fail to reach REMs, when they do, these programs are effective and well accepted, regardless of whether they are culturally adapted [15,18]. In fact, emerging research suggests that culturally adapted DMHIs do not outperform their original counterparts [68], raising questions about the need to undergo this complex, time-consuming, and resource-intensive process [15,18]. Therefore, future DMHI studies must collect information on who accesses these tools, engages with the content, and benefits from the intervention, with an emphasis on identifying real inequities (eg, limited uptake among Black individuals, poor engagement among Latinx individuals, more attenuated reductions in symptoms among Asian individuals) and using that data to inform precise adaptations as needed [15,39]. In traditional mental health services, this data-driven approach to culturally adapting treatments has proven more effective than assuming a priori adaptations are needed [69].

An important caveat regarding the effectiveness of non-culturally adapted DMHIs for REMs is that the Mind-Us program still leveraged previously proposed strategies to improve the cultural robustness of DMHIs through human support [18]. During the onboarding procedure, study staff discussed potential perceived mismatches between the content of the DMHI and the participant’s cultural background and identity, provided psychoeducation about mindfulness and DMHIs, and helped the participant see how mindfulness could be helpful in dealing with discrimination-related stressors. Since few researchers and clinicians are in a position to culturally adapt existing DMHIs or design new ones for specific REM groups, using human support to improve the cultural fit of DMHIs represents an alternative with significant implementation and dissemination potential. Onboarding procedures, weekly check-ins, and wrap-up strategies could be used as a flexible, idiographic, and personalized approach to address the current shortcomings of commercially available DMHIs when used with REM populations [18,70]. The potential of this approach is supported by numerous studies showing that DMHIs with some form of human support have better engagement [71] and larger treatment effects [72] compared with completely self-guided DMHIs. Regardless of their promise, human support models need to be further investigated through dismantling studies to determine which components of these implementation strategies drive their effects (eg, technical troubleshooting, motivational strategies, or discussions of cultural fit).

In contrast with the poor uptake found in previous app-based DMHI trials and real-world cases, we found a promising adoption of this program. This DMHI had an uptake rate of 100%, which contrasts with average uptake rates of 45% in RCTs [24] and 21% in real-world settings [25]. We attribute this success to the short onboarding call at the beginning of the intervention, in which participants were assisted in downloading the app, learning how to use it, and troubleshooting any technical issues. In designing this onboarding procedure, we followed recommendations to address common barriers faced by REMs when using DMHIs [18]. The effectiveness of onboarding procedures is supported by a previous study showing that a similar approach was effective in addressing barriers related to digital literacy and promoting DMHI uptake among REMs [73]. Thus, onboarding procedures that address issues with digital literacy show promise to promote uptake among REMs using DMHIs. However, this interpretation needs to be considered cautiously, given that our study design did not allow us to determine whether this high uptake was truly driven by the onboarding procedure or some other aspect or combination of factors in this DMHI program.

Participants in the DMHI group also used the app to complete approximately 1 session a day for a total of 1 hour of meditation per week for 4 weeks, reporting high program satisfaction. This consistent engagement and high program satisfaction throughout the program are significantly different from app usage patterns found in previous RCTs, where users only use the app a couple of times early in treatment [24], and app usage in the real world, where only 4% of participants continue using these digital tools after 2 weeks [26]. Similarly, 25% of participants in RCTs [24] and 96% of real-world app users [26] drop out after 4 weeks. Engagement in DMHI is crucial because it is often associated with better treatment outcomes [15]. These consistent engagement, low dropout, and high treatment satisfaction are especially encouraging because REMs face significant barriers to engaging with face-to-face mental health services [17,74]. Considering recent work showing that DMHI users also face similar barriers to those of traditional mental health services [27,28], this program shows promise to circumvent these problems, allowing REMs to engage in and benefit from mental health services via DMHIs. Despite our inability to infer the exact factors driving these effects, we believe that the use of multiple engagement-enhancement strategies, such as daily text messages [40], an onboarding procedure with study staff [18], and a short program length [40] could have contributed to these outcomes. Among these strategies, the onboarding procedure might have been especially beneficial for REMs, as participants problem-solved issues that could interfere with their everyday use of the app in advance. The potential effectiveness of providing support at the beginning of DMHI programs aligns with studies showing that app-based DMHIs with some type of human guidance tend to have better engagement and lower dropout compared with fully self-guided DMHIs [24]. Future studies are needed to determine the most effective engagement-enhancement strategies (eg, human support vs automated reminders), the mechanisms by which these strategies affect engagement and dropout (eg, self-efficacy), factors promoting program satisfaction (eg, therapeutic alliance with human supporter), and the specific cost-benefit of supplementing DMHIs via human support (eg, fully self-guided DMHIs being cheaper but leading to significantly smaller treatment effect sizes than guided DMHIs).

### Limitations and Future Directions

Findings in this study should be interpreted in the context of existing limitations. First, this study was conducted from 2020 to 2022, during the COVID-19 pandemic, nationwide lockdowns, and a social and political environment in the United States that made discrimination against REM groups more common and prominent. This unique intersection of conditions was likely to influence participants’ mental health needs, willingness to participate in this trial, and motivation to stay engaged with the DMHI. Another limitation is that this RCT did not include a measure of discrimination-based distress as an outcome. Instead, we considered high exposure to discrimination as a “risk factor” associated with increases in mental health symptoms [3]. While enrolling “at risk” samples is a common practice in DMHI research, we recognize that discrimination-based distress may be a meaningful clinical outcome over and above stress, anxiety, and depression. Although our sample was uniquely diverse in terms of race and ethnicity, participants were still mostly young, highly educated, English-speaking females. DMHI research to date has been dominated by these types of samples [15], and additional efforts are needed to recruit a more diverse set of participants across age, socioeconomic status, language preference, and gender. For instance, partnering with trusted community members (eg, pastors) and key organizations (eg, schools) [15] and using recruitment materials that directly target populations underrepresented in DMHI research [75] have proven effective strategies for engaging more diverse populations in DMHI trials. In this study, we also relied on participants’ willingness to share app usage data with the research team, and future work can benefit from obtaining app usage data directly from DMHIs to obtain more accurate estimates of treatment engagement. Similarly, information on participants’ reasons for stopping the use of the DMHI was not collected. Future studies examining the experiences of REM users qualitatively are warranted to better understand DMHI engagement and attrition among these groups. Even though our sample size and the number of assessments were similar to previous app-based mindfulness DMHI trials [22], future studies with larger sample sizes and long-term follow-ups are necessary to replicate these results, potentially identify smaller treatment effect sizes, and elucidate the duration of treatment effects. A final limitation is the lack of an active control condition, as none of the participants randomized to TAU received mental health services during the duration of the trial, effectively making this control condition a waitlist. Although considering the current state of the literature in app-based mindfulness DMHIs for REMs may minimize this concern [22,41], active control conditions are necessary to advance both mindfulness and DMHI effectiveness research.

### Conclusions

This study shows that self-guided DMHIs that combine commercially available digital tools with some human support and automated text messages hold significant promise to advance mental health equity for REMs. Among participants who received this non-culturally adapted DMHI, 48%‐61% experienced a clinically significant reduction in symptoms by the end of treatment, suggesting that low-intensity DMHIs can improve the well-being of REMs exposed to discrimination. Futher supporting the promise of this program, all participants accessed the DMHI, used it consistently throughout 4 weeks, reported high treatment satisfaction, and only a small number left the intervention prematurely. Crucially, the dissemination and implementation potential of this approach is substantial, considering the recent introduction of new Centers for Medicare and Medicaid Services codes (ie, G0552, G0553, and G0554) that has made both DMHIs and the human support necessary for their deployment eligible for insurance reimbursement, effectively creating the financial infraestructure necessary for the long-term viability of hybrid models of care in the United States.

## Supplementary material

10.2196/84328 Multimedia Appendix 1 Missing data analytic plan.

10.2196/84328 Checklist 1 CONSORT-eHEALTH checklist (V 1.6.1).

## Abbreviations

CONSORT-EHEALTH: Consolidated Standards of Reporting Trials of Electronic and Mobile Health Applications and Online Telehealth
DMHI: digital mental health intervention
FDR: false discovery rate
GAD-7: Generalized Anxiety Disorder-7
HIPAA: Health Insurance Portability and Accountability Act
IRRS-B: Index of Race-Related Stress-Brief
MCID: minimal clinically significant difference
MDM: Multicultural Discrimination Module
PHQ-8: Patient Health Questionnaire-8
PHQ-9: Patient Health Questionnaire-9
PSS: Perceived Stress Scale
RCT: randomized controlled trial
REM: racially and ethnically minoritized individual
STTS-R: Satisfaction with Therapy and Therapist Scale-Revised
TAU: treatment-as-usual
UCLA: University of California, Los Angeles
