# Supplementary material for: A Self-Guided App-Based Mindfulness Intervention for Racially and Ethnically Minoritized Individuals Who Experience Discrimination-Related Mental Health Symptoms: Randomized Controlled Trial
Source: J Med Internet Res. 2026 May 14;28:e84328. doi: 10.2196/84328 (PMC13175143; doi:10.2196/84328)
Supplement: Multimedia Appendix 1 [file jmir-v28-e84328-s001.docx]

**Mind-Us: Missing Data Appendix**

In this section, we report the primary result under a variety of missing data models to evaluate the sensitivity of the result to assumptions about the missing data mechanism.

**Missing Data Patterns**

Of the 155 participants, 13 were missing outcome data during at least one timepoint. No participants were missing on baseline data. Nine participants did not return for the 1^st^ follow-up, meaning they were missing scores on all outcomes at this timepoint, of these only 1 returned for the 2^nd^ follow-up. An additional 4 participants did not return for the 2^nd^ follow-up. There were no participants who partially completed the outcomes, meaning that if they were missing on one outcome they were missing all outcomes at that time point. This leads to three unique missing data patterns: OOO (N = 142), OMM (N = 8), OMO (N = 1), and OOM (N = 4), where the O indicates observed values and M indicates missing.

We wanted to explore potential factors which might predict missingness, including treatment condition, age, sex, and financial need. Twelve participants with missing data (92%) were in the treatment group, compared to 68 (48%) participants with complete data. The average age of those with missing data was 24.38 while the average age of those with complete data was 27.55. Twelve of the participants with missing data (92%) were female and one was male; whereas 116 participants with complete data (82%) were female, 21 were male, and 5 were non-binary. Two individuals with missing data (15%) indicated not having enough money to cover their monthly expenses, and 22 individuals with complete data (15%) indicated not having enough money to cover their monthly expenses.

**Missing Complete at Random (MCAR)**

A missing completely at random mechanism would mean that no variables in the dataset or the missing value itself predict whether or not an individual is missing. Little’s test of MCAR on the wide-form data was not statistically significant ($\chi^{2}\left( 40 \right)=38.7$, $p= .53$) suggesting there is not sufficient evidence to reject the null hypothesis of MCAR.

Analyses using list-wise deletion on the long form data, aligned with data which is MCAR, show a significantly greater decrease in depression in the treatment group compared to control for baseline to follow-up 1 (b = -1.84, se = 0.78, t(284) = -2.36, p = .02) and baseline to follow-up 2 (b = -2.92, se = 0.90, t(284) = -3.26, p = .001). The difference between treatment and control in change in depression between follow-up 1 and follow-up 2 was not statistically significant (b = -1.08, se = .79, t(284) = -1.37, p = .17).

The same analyses were conducted for stress and anxiety. The results showed a significantly greater decrease in stress in the treatment group compared to control for baseline to follow-up 1 (b = -2.46, se = 0.88 , t(284) = -2.78, p = .006), baseline to follow-up 2 (b = -4.47, se = 1.04, t(284) = -4.32, p < .0001), and follow-up 1 to follow-up 2 (b = -2.01, se = 0.90, t(284) = -2.24, p = .03). There was also a significantly greater decrease in anxiety in the treatment group compared to control for baseline to follow-up 1 (b = 2.36, se =0.74 , t(284) = -3.17, p = .002) and baseline to follow-up 2 (b = -3.40, se = 0.83, t(284) =-4.11, p = .0001), but not follow-up 1 to follow-up 2 (b = -1.05, se = 0.76, t(284) = -1.38, p = 17).

While these model results align with the results in the primary analysis, and the test of MCAR was not statistically significant, we caution readers from overinterpreting such a simplistic analysis of the current data. Given the relatively small sample size of the study, the test of MCAR may not be well powered. Observationally, there are some strong predictors of missingness within the data, suggesting a missing at random mechanism, and given the nature of the study drop out due to poor mental health outcomes seems likely, suggesting a missing not at random mechanism. Both approaches are explored next.

**Missing at Random (MAR)**

For MAR models, the assumption is that observed variables predict the missingness in the observation but the missing value itself does not predict the missingness. For both the MAR models and the Missing Not at Random (MNAR) models (presented below) we use a shared parameter model, where a random intercept is shared across the models of the outcome at baseline, first follow-up, and second follow-up. The outcome at each timepoint is a function of this shared parameter, treatment condition, sex, age, and whether the participant had enough money to cover their monthly expenses at baseline. Additionally, the outcome at first follow-up is predicted by the outcome at baseline, and the outcome at second follow-up is predicted by the outcome at first follow-up, to align with the AR1 structure of the analysis model. We fit three types of MAR models: separate outcomes, correlated shared parameters, and single shared parameter. Each of these models is described below.

***Separate Outcome Models***

The separate outcome model is fit separately for depression, stress, and anxiety and can be expressed as follows:

$$Y_{0i}=\theta_{i}+b_{01}T_{i}+b_{02}S_{1i}+b_{03}S_{2i}+b_{04}A_{i}+b_{05}E_{i}+e_{0i}$$

Where $Y_{0i}$ is the outcome (stress, depression, or anxiety) at baseline, $\theta_{i}$ is a normally distributed random intercept, and $T_{i}$, $A_{i}$, and $E_{i}$ are treatment condition, age, and whether or not individuals had enough money at baseline. $S_{1i}$ and $S_{2i}$ are dummy codes indicating sex of person $i$. In this an all models to follow all covariates ($T_{i}$, $A_{i}$, $E_{i}$, $S_{1i}$, and $S_{2i}$) are grand mean centered. Note that the baseline model does not have a fixed intercept, meaning the mean of $\theta$ will capture the baseline mean of the outcome $Y$. The model for the first follow-up can be expressed as:

$$Y_{1i}=b_{10}+\theta_{i}+b_{11}T_{i}+b_{12}S_{1i}+b_{13}S_{2i}+b_{14}A_{i}+b_{15}E_{i}+b_{lag}Y_{0i}+e_{1i}$$

In the model for $Y_{1i}$, the outcome at first follow-up, there is a fixed intercept ($b_{10}$) representing the deviation of the mean at first follow-up from the baseline mean of the outcome. $\theta$ allows for the random variability of individuals around that mean, but it does not have a coefficient (i.e., its coefficient is fixed at 1) to ensure $\theta$ contributes equally to the outcome in each model. The other predictors are the same for follow-up except baseline levels of the outcome $Y_{0i}$ are included with a coefficient $b_{lag}$ which will appear in the model for second follow-up as well. Finally, the model for second follow-up can be expressed as:

$$Y_{2i}=b_{20}+\theta_{i}+b_{21}T_{i}+b_{22}S_{1i}+b_{23}S_{2i}+b_{24}A_{i}+b_{25}E_{i}+b_{lag}Y_{1i}+e_{2i}$$

This model for the outcome at second follow-up ($Y_{2i}$) follows a similar structure as the previous model, with a fixed intercept ($b_{20}$) and random intercept ($\theta$). The other predictors are the same, except the outcome at first follow-up ($Y_{1i}$) is included with a coefficient $b_{lag}$ which is fixed to be the same across the first and second follow-up models. In the separate outcome models, these equations are estimated simultaneously three times: once for depression, stress, and anxiety each, meaning the models do not share information across them.

***Correlated Shared Parameters***

The correlated shared parameter model can be expressed similarly, where now we will use $Y$ to represent depression, $Z$ to represent stress, and $W$ to represent anxiety. The equations are as follows:

$$Y_{0i}=\theta_{Yi}+b_{Y01}T_{i}+b_{Y02}S_{1i}+b_{Y03}S_{2i}+b_{Y04}A_{i}+b_{Y05}E_{i}+e_{Y0i}$$

$$Y_{1i}=b_{Y10}+\theta_{Yi}+b_{Y11}T_{i}+b_{Y12}S_{1i}+b_{Y13}S_{2i}+b_{Y14}A_{i}+b_{Y15}E_{i}+b_{Ylag}Y_{0i}+e_{Y1i}$$

$$Y_{2i}=b_{Y20}+\theta_{Yi}+b_{Y21}T_{i}+b_{Y22}S_{1i}+b_{Y23}S_{2i}+b_{Y24}A_{i}+b_{Y25}E_{i}+b_{Ylag}Y_{1i}+e_{Y2i}$$

$$Z_{0i}=\theta_{Zi}+b_{Z01}T_{i}+b_{Z02}S_{1i}+b_{Z03}S_{2i}+b_{Z04}A_{i}+b_{Z05}E_{i}+e_{Z0i}$$

$$Z_{1i}=b_{Z10}+\theta_{Zi}+b_{Z11}T_{i}+b_{Z12}S_{1i}+b_{Z13}S_{2i}+b_{Z14}A_{i}+b_{Z15}E_{i}+b_{Zlag}Z_{0i}+e_{Z1i}$$

$$Z_{2i}=b_{Z20}+\theta_{Zi}+b_{Z21}T_{i}+b_{Z22}S_{1i}+b_{Z23}S_{2i}+b_{Z24}A_{i}+b_{Z25}E_{i}+b_{Zlag}Z_{1i}+e_{Z2i}$$

$$W_{0i}=\theta_{Wi}+b_{W01}T_{i}+b_{W02}S_{1i}+b_{W03}S_{2i}+b_{W04}A_{i}+b_{W05}E_{i}+e_{W0i}$$

$$W_{1i}=b_{W10}+\theta_{Wi}+b_{W11}T_{i}+b_{W12}S_{1i}+b_{W13}S_{2i}+b_{W14}A_{i}+b_{W15}E_{i}+b_{Wlag}W_{0i}+e_{W1i}$$

$$W_{2i}=b_{W20}+\theta_{Wi}+b_{W21}T_{i}+b_{W22}S_{1i}+b_{W23}S_{2i}+b_{W24}A_{i}+b_{W25}E_{i}+b_{Wlag}W_{1i}+e_{W2i}$$

In this model, $\theta_{Y}$, $\theta_{Z}$, and $\theta_{W}$ are normally distributed random effects which have estimated means and variances and are allowed to be correlated with each other. This estimation procedure is valuable because it allows for estimation of how much an individual’s random intercept, on for example depression, is correlated with their random intercepts, for example on stress. This begs the question however, whether there is value is estimating each of these random effects separately, or constraining them to be the same random effect across outcomes, which is achieved in the single shared parameter model.

***Single Shared Parameter***

This model can be expressed as:

$$Y_{0i}=\boldsymbol{b}_{\boldsymbol{Y}\boldsymbol{00}}\boldsymbol{+}\boldsymbol{b}_{\boldsymbol{Y}}\boldsymbol{\theta}_{\boldsymbol{i}}+b_{Y01}T_{i}+b_{Y02}S_{1i}+b_{Y03}S_{2i}+b_{Y04}A_{i}+b_{Y05}E_{i}+e_{Y0i}$$

$$Y_{1i}=b_{Y10}+\boldsymbol{b}_{\boldsymbol{Y}}\boldsymbol{\theta}_{\boldsymbol{i}}+b_{Y11}T_{i}+b_{Y12}S_{1i}+b_{Y13}S_{2i}+b_{Y14}A_{i}+b_{Y15}E_{i}+b_{Ylag}Y_{0i}+e_{Y1i}$$

$$Y_{2i}=b_{Y20}+\boldsymbol{b}_{\boldsymbol{Y}}\boldsymbol{\theta}_{\boldsymbol{i}}+b_{Y21}T_{i}+b_{Y22}S_{1i}+b_{Y23}S_{2i}+b_{Y24}A_{i}+b_{Y25}E_{i}+b_{Ylag}Y_{1i}+e_{Y2i}$$

$$Z_{0i}=\boldsymbol{b}_{\boldsymbol{Z}\boldsymbol{00}}\boldsymbol{+}\boldsymbol{b}_{\boldsymbol{Z}}\boldsymbol{\theta}_{\boldsymbol{i}}+b_{Z01}T_{i}+b_{Z02}S_{1i}+b_{Z03}S_{2i}+b_{Z04}A_{i}+b_{Z05}E_{i}+e_{Z0i}$$

$$Z_{1i}=b_{Z10}+\boldsymbol{b}_{\boldsymbol{Z}}\boldsymbol{\theta}_{\boldsymbol{i}}+b_{Z11}T_{i}+b_{Z12}S_{1i}+b_{Z13}S_{2i}+b_{Z14}A_{i}+b_{Z15}E_{i}+b_{Zlag}Z_{0i}+e_{Z1i}$$

$$Z_{2i}=b_{Z20}+\boldsymbol{b}_{\boldsymbol{Z}}\boldsymbol{\theta}_{\boldsymbol{i}}+b_{Z21}T_{i}+b_{Z22}S_{1i}+b_{Z23}S_{2i}+b_{Z24}A_{i}+b_{Z25}E_{i}+b_{Zlag}Z_{1i}+e_{Z2i}$$

$$W_{0i}=\boldsymbol{b}_{\boldsymbol{W}\boldsymbol{00}}\boldsymbol{+}\boldsymbol{b}_{\boldsymbol{W}}\boldsymbol{\theta}_{\boldsymbol{i}}+b_{W01}T_{i}+b_{W02}S_{1i}+b_{W03}S_{2i}+b_{W04}A_{i}+b_{W05}E_{i}+e_{W0i}$$

$$W_{1i}=b_{W10}+\boldsymbol{b}_{\boldsymbol{W}}\boldsymbol{\theta}_{\boldsymbol{i}}+b_{W11}T_{i}+b_{W12}S_{1i}+b_{W13}S_{2i}+b_{W14}A_{i}+b_{W15}E_{i}+b_{Wlag}W_{0i}+e_{W1i}$$

$$W_{2i}=b_{W20}+\boldsymbol{b}_{\boldsymbol{W}}\boldsymbol{\theta}_{\boldsymbol{i}}+b_{W21}T_{i}+b_{W22}S_{1i}+b_{W23}S_{2i}+b_{W24}A_{i}+b_{W25}E_{i}+b_{Wlag}W_{1i}+e_{W2i}$$

In the equations above the elements which are changed from the correlated shared parameter model are bolded. Importantly, $\theta_{i}$ is fixed to be normally distributed with a mean of zero and variance of 1, and is included in all the models of $Y$, $Z$, and $W$. To allow the mean of depression, stress, and anxiety to differ, intercepts are introduced in the baseline equations where they were not included in the previous model. In the previous models the means were allowed to differ by allowing for different random intercepts to each have unique means. In addition, the coefficients $b_{Y}$, $b_{Z}$, and $b_{W}$ are introduced as coefficients for $\theta$ allowing for outcome specific variances.

***Results***

Twenty datasets are imputed using the above models. For all models we used a 10,000-sample burn-in period, 20 chains, 10,000 iterations per chain, and 20 imputed datasets using the BLIMP. Code is publicly available at https://github.com/Quantitative-Research-Collaboratory/Mind-Us-Intervention. We fit this model separately for each outcome: depression, stress, and anxiety. Additionally, we fit this model simultaneously for each outcome with three correlated shared parameters, and again with a single shared parameter. Results of the main results for each MAR model are presented in Table below.

Performance metrics for the separate and correlated-shared models were good (PSR < 1, Effective N > 100). However, for the single shared parameter model performance metrics suggested the model was unstable. For example, the effective N for the correlation between the shared parameter and different outcomes and its coefficients were too low (e.g., 10 - 40). This suggests that caution should be used in interpreting this model, though we report it here for completeness as it is a candidate model in the MNAR section below as well.

Overall, the results of the models were very consistent with each other: Depression and anxiety saw greater reduction for the treatment group from baseline to first follow-up and baseline to second follow-up, but not first follow-up to second follow-up. Whereas stress saw significantly greater reduction for the treatment group from baseline to first follow-up, baseline to second follow-up, and first to second follow-up. These results are very promising as they are consistent with the primary analysis presented in the manuscript and suggest that the results are not sensitive to the different choices of missingness models explored here.

| **MAR Models** | Separate | | | | Correlated Shared | | | | Single Shared | | | |
| --- | --- | --- | --- | --- | --- | --- | --- | --- | --- | --- | --- | --- |
|  | b | se | t | p | b | se | t | p | b | se | t | p |
| Depression | | | | | | | | | | | | |
| Baseline vs. F1 | -1.83 | 0.78 | -2.34 | 0.02 | -1.75 | 0.77 | -2.26 | .02 | -1.86 | 0.78 | -2.38 | 0.02 |
| Baseline vs. F2 | -2.94 | 0.92 | -3.21 | .001 | -2.81 | 0.89 | -3.17 | .002 | -2.84 | 0.88 | -3.23 | 0.001 |
| F1 vs. F2 | -1.11 | 0.80 | -1.39 | .16 | -1.06 | 0.78 | -1.36 | .17 | -0.99 | 0.78 | -1.26 | 0.21 |
| Stress | | | | | | | | | | | | |
| Baseline vs. F1 | -2.45 | 0.89 | -2.76 | .006 | -2.39 | 0.89 | -2.67 | .008 | -2.41 | 0.89 | -2.72 | 0.006 |
| Baseline vs. F2 | -4.49 | 1.04 | -4.32 | < .001 | -4.47 | 1.03 | -4.33 | <.001 | -4.52 | 1.03 | -4.40 | <.001 |
| F1 vs. F2 | -2.04 | 0.91 | -2.26 | .02 | -2.09 | 0.92 | -2.28 | .02 | -2.11 | 0.912 | -2.32 | 0.02 |
| Anxiety | | | | | | | | | | | | |
| Baseline vs. F1 | -2.38 | 0.76 | -3.16 | .002 | -2.32 | 0.75 | -3.09 | .002 | -2.31 | 0.75 | -3.10 | 0.002 |
| Baseline vs. F2 | -3.40 | 0.84 | -4.06 | <.001 | -3.35 | 0.81 | -4.14 | <.001 | -3.31 | 0.80 | -4.13 | <.001 |
| F1 vs. F2 | -1.02 | 0.77 | -1.32 | 0.186 | -1.03 | 0.75 | -1.37 | .171 | -1.00 | 0.75 | -1.338 | 0.18 |

**Missing Not at Random (MNAR)**

Because the study is a clinical trial is it not unreasonable to assume that an individual’s current mental health (depression, stress, and anxiety) might impact whether that individual reports these variables on a given day. This would suggest a missing not at random (MNAR) mechanism.

We fit several models to explore missing not at random mechanisms. The primary model presented in the manuscript uses a single latent variable with unique mean and variance for each outcome (depression, stress, and anxiety) and each outcome has a unique lag parameter. We modelled missingness at Time 2 as predicted by the single latent variable, condition, and economic need.

The models correspond to the three types of models fit for MAR: separate models, correlated latent variables across the three outcomes, or a single shared latent variable across the three outcomes. The same fit specifications as the MAR models were used (e.g., 10,000 burn-in).

Performance metrics for the depression only model we largely good (PSR < 1.1, Effective N > 100), except the effective N in the missingness model were relatively low (Intercept = 37, random intercept = 32, treatment group = 63). Performance metrics for the stress-only model we largely good (PSR < 1.1, Effective N > 100) with some key exceptions: the effective N (62) and PSR (1.253) for the random intercept variance were poor, and the intercepts and lag parameters for the models of stress at first and second follow-up had high PSR and low effective N (PSR <= 1.303, N_eff >= 53). Performance metrics for the anxiety only model we largely good (PSR < 1.1, Effective N > 100) with some key exceptions: the effective N (70) and PSR (1.179) for the random intercept variance were poor; the intercepts and lag parameters for the models of anxiety at first and second follow-up had high PSR and low effective N (PSR <= 1.193, N_eff >= 68); and the intercept and random intercept coefficient had high PSR (PSR <=1.256) and low effective N (N_eff >=64). Performance metrics for the correlated shared parameter models were generally good (PSR < 1.1, Effective N > 100) with one exception: The PSR and effective N for the random intercepts for depression and anxiety predicting missingness were poor (PSR <= 1.353, N_eff >= 45). This is likely because the random intercepts are highly correlated ($r_{anx,dep}= .97$, $r_{anx, str}=0.93$, $r_{dep,str}=0.87$). The high correlation among the random intercepts suggests the validity of the model with a single random intercept shared across all three outcomes. Performance metrics for this model were generally very good, except the PSR and effective N were very poor for the outcome specific scaling parameters ($b_{Y}$, $b_{Z}$, and $b_{W}$; PSR <= 9.73, N_eff >= 10).

| **MNAR Models** | Separate | | | | Correlated Shared | | | | Single Shared | | | |
| --- | --- | --- | --- | --- | --- | --- | --- | --- | --- | --- | --- | --- |
|  | b | se | t | p | b | se | t | p | b | se | t | p |
| Depression | | | | | | | | | | | | |
| Baseline vs. F1 | -1.83 | 0.80 | -2.29 | 0.02 | -1.83 | 0.77 | -2.37 | 0.02 | -1.79 | 0.78 | -2.31 | .02 |
| Baseline vs. F2 | -3.03 | 0.93 | -3.26 | 0.001 | -2.93 | 0.88 | -3.35 | .001 | -2.80 | 0.88 | -3.18 | .001 |
| F1 vs. F2 | -1.20 | 0.80 | -1.51 | 0.13 | -1.10 | 0.79 | -1.40 | 0.16 | -1.01 | 0.77 | -1.30 | 0.19 |
| Stress | | | | | | | | | | | | |
| Baseline vs. F1 | -2.41 | 0.90 | -2.89 | .007 | -2.42 | 0.90 | -2.70 | .007 | -2.30 | 0.89 | -2.57 | 0.01 |
| Baseline vs. F2 | -4.54 | 1.06 | -4.30 | <.001 | -4.47 | 1.05 | -4.28 | <.001 | -4.47 | 1.04 | -4.31 | <.001 |
| F1 vs. F2 | -2.13 | 0.90 | -2.36 | 0.018 | -2.05 | 0.92 | -2.25 | 0.025 | -2.18 | 0.91 | -2.39 | 0.02 |
| Anxiety | | | | | | | | | | | | |
| Baseline vs. F1 | -2.40 | 0.76 | -3.14 | 0.002 | -2.24 | 0.75 | -2.98 | .003 | -2.35 | 0.74 | -3.18 | 0.001 |
| Baseline vs. F2 | -3.47 | 0.86 | -4.04 | <.001 | -3.32 | 0.81 | -4.09 | <.001 | -3.32 | 0.81 | -4.12 | <.001 |
| F1 vs. F2 | -1.07 | 0.76 | -1.41 | 0.16 | -1.08 | 0.74 | -1.46 | .144 | -0.98 | 0.75 | -1.30 | 0.19 |

**Model Comparisons**

There are six candidate modeling approaches we considered: MAR vs. MNAR combined with separate, correlated, or shared parameter models. We used a combination of fit metrics to evaluate the quality of fit of each of these models. The separate models were not comparable to the other two types of models (correlated and shared parameter) because they do not incorporate all information across all variables. However, comparing the MAR to MNAR models for separate outcomes, the MAR model always fit better than the MNAR model. Comparing the models that examined all outcomes together, the MAR model with the single shared parameter fit best. Thus, this is the model we reported in the primary manuscript.

Model Fit Table

| Missingness Mechanism | Model | Outcome | Marginal DIC2 | Marginal WAIC | Variance in Missingness Explained [95% Credible Interval] |
| --- | --- | --- | --- | --- | --- |
| MAR | Separate | *Depression* | *3424.741* | *3496.411* | *62.2% [50.7%, 71.3%]* |
|  |  | *Stress* | *3503.516* | *3572.189* | *60.6% [48.3%, 70.6%]* |
|  |  | *Anxiety* | *3293.696* | *3362.457* | *53.7% [39.3%, 64.3%]* |
| MAR | Correlated Shared | All | 9439.531 | 9605.930 | 61.5% [52.2%, 69.6%] |
| **MAR** | **Single Shared** | **All** | **8165.279** | **8261.262** | **58.7% [50.0%,66.2%]** |
| MNAR | Separate | Depression | 3897.728 | 4015.092 | 37.0% [9.8%,74.4%] |
|  |  | Stress | 3983.004 | 4097.131 | 29.8% [7.5%,56.7%] |
|  |  | Anxiety | 3792.522 | 3865.257 | 35.6%  [9.2%,79.2%] |
| MNAR | Correlated Shared | All | 9996.629 | 10193.439 | 83.6%  [30.2%, 96.1%] |
| MNAR | Single Shared | All | 8648.303 | 8790.430 | 29.1%  [7.4%, 54.8%] |

**Bold** indicates “best model” for all outcomes simultaneously. *Italics* indicates “best model” for separate outcomes.

**Engagement Data**

Of the 80 participants in the DMHI group, nine were missing engagement data because they did not share their app usage information with the research team. Participants who shared their app usage data showing no engagement with the DMHI (i.e., 0 days of use, 0 meditations completed, 0 minutes meditated) were not considered to have missing data. At mid-treatment, two participants had no engagement with the DMHI but shared app usage data. At post-treatment, six participants had no engagement with the DMHI but shared app usage data. During the 28-day duration of the intervention in total, only two participants had no engagement with the DMHI but shared app usage data.

First, we examined all engagement metrics only among participants who shared their app usage data regardless of their level of engagement with the DMHI. Among those 71, participants used the app mean 9.06 days (*SD*=3.6; range=0-14), completed mean 13.48 meditations (*SD*=8; range=0-52), and meditated mean 116.56 minutes (*SD*=94.7; range=0-652) from baseline to mid-treatment. From mid-treatment to post-treatment, participants used the app mean 7.94 days (*SD*=4.7; range=0-14), completed mean 11.83 meditations (*SD*=18.3; range=0-156), and meditated mean 130.07 minutes (*SD*=189.7; range=0-1543). During the 28-day intervention in total, participants used the app mean 16.97 days (*SD*=7.9; range=0-28), completed mean 25.31 meditations (*SD*=24.71; range=0-208), and meditated mean 246.63 minutes (*SD*=274.9; range=0-2195).

We then examined all engagement metrics only among participants who used the DMHI at least once during the 28-day program and shared their app usage data with the research team. Among those 69, participants used the app mean 9.32 days (*SD*=3.4; range=1-14), completed mean 13.87 meditations (*SD*=7.8; range=1-52), and meditated mean 119.94 minutes (*SD*=94; range=1-652) from baseline to mid-treatment. From mid-treatment to post-treatment, participants used the app mean 8.17 days (*SD*=4.6; range=1-14), completed mean 12.17 meditations (*SD*=18.4; range=1-156), and meditated mean 133.84 minutes (*SD*=191.1; range=1-1543). During the 28-day intervention in total, participants used the app mean 17.46 days (*SD*=7.5; range=1-28), completed mean 26.04 meditations (*SD*=24.68; range=1-208), and meditated mean 253.78 minutes (*SD*=275.6; range=0-2195).

Similarly, we examined all engagement metrics under a conservative assumption that the nine participants who did not share app usage data did not use the app at all during the 28-day program. Among all 80, participants used the app mean 8.04 days (*SD*=4.5; range=0-14), completed mean 11.96 meditations (*SD*=8.7; range=0-52), and meditated mean 103.45 minutes (*SD*=96.6; range=0-652) from baseline to mid-treatment. From mid-treatment to post-treatment, participants used the app mean 7.05 days (*SD*=5.1; range=0-14), completed mean 10.50 meditations (*SD*=17.6; range=0-156), and meditated mean 115.44 minutes (*SD*=183.2; range=0-1543). During the 28-day intervention in total, participants used the app mean 15.06 days (*SD*=9.2; range=0-28), completed mean 22.46 meditations (*SD* =24.61; range=0-208), and meditated mean 218.89 minutes (*SD* = 270.4; range = 0-2195).

Finally, we examined all engagement metrics with an imputed dataset under a MAR missing data assumption with single-shared parameters consistent with the best fitting model observed for missing outcome data. Imputed values were allowed to be 0 to capture that some participants might have downloaded the DMHI but not used it at all during the 28-day program. These are the engagement outcomes reported in the manuscript. Among those 80, participants used the app mean 9.01 days (*SD*=3.6; range=0-14), completed mean 13.27 meditations (*SD*=7.7; range=0-52), and meditated mean 113.76 minutes (*SD*=90.5; range=0-652) from baseline to mid-treatment. From mid-treatment to post-treatment, participants used the app mean 7.85 days (*SD*=4.7; range=0-14), completed mean 11.58 meditations (*SD*=17.2; range=0-156), and meditated mean 124.90 minutes (*SD*=179.7; range=0-1543). During the 28-day intervention in total, participants used the app mean 16.83 days (*SD*=7.8; range=0-28), completed mean 24.85 meditations (*SD*=23.3; range=0-208), and meditated mean 238.67 minutes (*SD*=260.7; range=0-2195).
